# Brown seaweed (AquaArom) supplementation increases food intake and improves growth, antioxidant status and resistance to temperature stress in Atlantic salmon, *Salmo salar*

**DOI:** 10.1371/journal.pone.0219792

**Published:** 2019-07-15

**Authors:** Collins Kamunde, Ravinder Sappal, Tarek Mostafa Melegy

**Affiliations:** Department of Biomedical Sciences, Atlantic Veterinary College, University of Prince Edward Island, PE, Canada; University of Illinois, UNITED STATES

## Abstract

Seaweeds represent a vast resource that remains underutilized as an ingredient in aquafeeds. Here we probed the effect of addition of AquaArom, a seaweed meal derived from brown seaweeds (Laminaria sp., kelp), to fish feed on growth performance, antioxidant capacity and temperature responsiveness of mitochondrial respiration. A commercial salmonid feed was mixed with 0 (control), 3, 6 and 10% seaweed and fed to Atlantic salmon (*Salmo salar*) smolts for 30 days. The smolts consumed more of the seaweed-supplemented food relative to the control and there were no mortalities. Compared with the control, the final fish weight, standard length, weight gain and SGR were higher in fish fed diets supplemented with the 3 and 10% seaweed, while growth performance for fish maintained on 6% seaweed remained neutral. Importantly, seaweed supplementation increased protein efficiency ratio (PER) and tended to improve food conversion ratio (FCR). Although the hepatosomatic and visceral indices did not change, whole gut and intestinal weights and lengths were higher in fish maintained on seaweed-supplemented diets suggesting increased retention time and a larger surface area for food digestion and nutrient absorption. Measurement of antioxidant status revealed that seaweed supplementation dose-dependently increased plasma total antioxidant capacity as well as the level of glutathione, and activities of catalase and superoxide dismutase in liver mitochondria. Moreover, seaweed supplementation reduced the effect of acute temperature rise on mitochondrial respiration and proton leak. Overall, these data suggest that AquaArom can be mixed with fish food up to 10% to increase food consumption and enhance growth performance, as well as to improve antioxidant capacity and alleviate adverse effects of stressors such as temperature in fish.

## Introduction

There is sustained interest in the use of plant ingredients in aquafeeds [1–4] but much remains unknown about the utility of aquatic macroalgae (seaweeds) in nutrition of aquatic animals. Although the use of terrestrial vegetable ingredients in aquafeeds, e.g., as fishmeal replacement, has been shown to negatively impact food digestibility, growth performance and the overall fish health status [2,3,5–7], the absence of negative effects has also been demonstrated [8–11]. Notably, better growth performance in fish fed low levels of vegetable material derived from the aquatic environment has been reported [8,10–12]. Because seaweeds are aquatic and a source of polyunsaturated fatty acids essential for fish growth [12], it is possible that they are more amenable to inclusion in aquafeeds than ingredients derived from terrestrial plants. Indeed, seaweeds are considered natural forage for fish but a major impediment to their use in aquafeeds is that they vary substantially in their biochemical and nutritional profiles according to species [4,12,13–16]. In part because of this variability and differences in feeding habits among fish, effects of seaweed supplementation in aquafeeds are highly variable. Generally, beneficial effects or absence of adverse effects have been observed at low inclusion levels of up to 10% for the majority of the seaweeds and fish species tested [6,12,14,17–19]. The effects of seaweed supplementation most relevant in aquaculture include stimulation of growth performance, enhancement of feed utilization efficiency, improvement of nutrient assimilation, and improvement of fatty acid profile (increase in long chain n-3 polyunsaturated fatty acids) in muscle [4,14,20–22]. In addition, seaweeds contain a wide array of bioactive compounds/secondary metabolites with potential utility as phytonutrients/nutraceuticals in animal feed [4,6,23,24]. There is much interest in knowing if these bioactive compounds can improve the overall health status of fish by enhancing resistance to disease, improving antioxidant capacity, and/or alleviating routine aquaculture stress associated with crowding or events such as handling (e.g., during grading and vaccination) and transportation [25–28].

Because at high inclusion levels seaweeds have been shown to impair fish growth performance and feed efficiency [4,14–17], determining the inclusion levels that improve fish growth performance and/or health status remains the primary focus of most of the studies. Such knowledge could permit the replacement of expensive ingredients of fish feed such as fishmeal and/or mixing of small amounts of seaweeds or their extracts with finished aquafeeds to harness the growth-unrelated beneficial effects. However, the variability in the biochemical composition and inconsistent effects of seaweed supplementation among fish necessitates testing the effects of supplementation of specific seaweed on specific fish species. In particular, salmonids are the most important aquaculture fish and use the greatest volumes of fishmeal and fish oil in aquafeeds [29]; thus demonstrating a role of seaweeds in salmonid nutrition would have major implications for aquaculture.

In the present study, we used Atlantic salmon smolts to test the effects of fortification of commercial fish food with AquaArom (a seaweed meal derived from brown seaweeds of the genus Laminaria) as opposed to replacement of a standard aquafeed ingredient, e.g., fishmeal. First, we determined the effect of AquaArom added to commercial salmonid food on food intake and growth performance of smolts. Based on related previous studies [14,28] and the notion that beneficial effects of seaweeds would result from the micronutrients they contain, we hypothesized that the concentration-growth response relationship would be biphasic with mid-level seaweed inclusion imposing the highest growth performance. Second, because effects of seaweeds in fish nutrition may manifest as favorable biological and health responses rather than as direct changes in growth performance, we assessed the effect of AquaArom supplementation on antioxidant capacity and response to acute thermal stress. Our hypothesis was that seaweed supplementation would increase exogenous circulating levels of antioxidants but reduce levels of endogenous antioxidant molecules and activities of antioxidant defence systems. Third, we tested the role of mitochondria in mediating the effects of AquaArom supplementation. Here, the hypothesis was that changes in growth performance and antioxidant status following seaweed supplementation would be reflected in mitochondrial function because mitochondria are regarded as the key sites for energy conversion and reactive oxygen species (ROS) regulation in a cell [30–32].

## Materials and methods

### Ethical considerations

The study and all of the experimental procedures that fish were subjected to were approved by the University of Prince Edward Island Animal Care Committee (protocol #16–026) consistent with the Canadian Council on Animal Care guidelines.

### Experimental diets

Brown seaweed flakes (AquaArom) prepared from Laminaria sp. (kelp) were provided by ADDiCAN Inc., Canada. Experimental diets were made in-house by supplementing finished commercial fish feed, EWOS micro crumble for salmonids (Ewos Canada Ltd, St. George, New Brunswick, Canada), with the required amount of seaweed calculated to deliver 0 (control), 3, 6, and 10% seaweed on a dry matter basis. The EWOS micro crumble contained, crude protein: 54% (minimum), crude fat: 16% (minimum), crude fiber: 1.3% (maximum), calcium: 2.5 (actual), phosphorous: 1.5% (actual), sodium: 0.5% (actual), vitamin A: 20,000 i.u. kg^−1^ (minimum), vitamin D3: 3000 i.u. kg^−1^ (minimum), and vitamin E: 400 i.u. kg^−1^ (minimum). The primary objective of our study was to identify the amount of seaweed that could be mixed with finished commercial salmonid feed resulting in beneficial effects as opposed to partially or completely replacing a dietary ingredient such as fishmeal. Briefly, the commercial salmonid feed and seaweed were ground and appropriate amounts were mixed to achieve the desired level of seaweed supplementation. Millipore water equivalent to 10% of diet weight was added and mixed in a pasta maker for 30 min to ensure homogenous distribution of the seaweed throughout the food. Thereafter, a further 30% diet weight Millipore water was added (bringing the total volume of water added to 40% diet weight) and mixed for a further 15 min. The food was then subsequently extruded via a round 3 mm disc, air-dried to constant weight, and broken into small pellets (approximately 3 mm) by hand. Control diet was processed in the same way except that no seaweed was added. The experimental diets were kept at -20°C till they were used in the feeding trial.

### Analysis of food and seaweed composition

Analysis of the composition of the experimental diets and seaweed was done at PEI Analytical Laboratory (https://www.princeedwardisland.ca/en/information/agriculture-and-fisheries/pei-analytical-laboratories-peial). The laboratory, operated by the Provincial Government of Prince Edward Island, is accredited to the international standard for the general requirements for competence of testing and/or calibration laboratories (ISO/IEC 17025:2005) by the Standards Council of Canada. Descriptions of the analytical methods that were used are provided in S1 Table.

### Feeding trial and sampling

The feeding trial was conducted at the Atlantic Veterinary College aquatic facility. Atlantic salmon smolts (initial average weight: 77 g) were obtained from Northern Harvest, Cardigan, PE, and were maintained in a 1200-L tank supplied with flow-through aerated well-water containing (mg/L): Na 47.1, Cl 137.3, Ca 58.8, Mg 27.6, hardness 260 (as CaCO_3_). The water pH and temperature were 7.5–8.0 and 10.5–11°C (nominal 11 ± 1 ^o^C), respectively. The smolts were acclimated to these laboratory conditions for 1 month and were fed 2% wet bw daily with 3.0 mm EWOS transfer for salmonids.

Following the acclimation period, the smolts (at this point their average weight was 92 g) were randomly distributed in groups of 8 in 160-L tanks comprising triplicates for each of the 4 experimental groups (control and 3, 6, and 10% seaweed supplementation). The tanks were then randomly distributed in a 2 × 6 block within the experimental room. A photoperiod of 12 h light:12 h dark was maintained. The fish were hand-fed the designated diet twice a day, once in the morning (08:00–09:00 h) and again in the evening (18:00–19:00 h) for 30 days. After dispensing the food into the tanks, fish were allowed to feed for 1 h following which the uneaten food was collected, dried to constant weight at 60 ^o^C. The amount of uneaten food was subtracted from the total amount of food dispensed to estimate food consumption per tank, food conversion ratio (FCR) and protein efficiency ratio (PER). Bulk fish weights obtained weekly were used to calculate the ration for the following week. On day 30 fish were euthanized with an overdose (300 mg/L) of MS-222 (Sigma-Aldrich Co., LLC, Bellefonte, USA), individually weighed and their standard lengths and body depth were measured; the body weight and length data were used to calculate condition factors (CF). Blood was obtained by caudal venipuncture and centrifuged at 10,000g to obtain plasma which was stored at -80 ^o^C for determination of total antioxidant capacity. The fish were then dissected to harvest livers and viscera which were weighed for calculation of hepatosomatic (HSI) and viscerosomatic (VSI) indexes, respectively. The weights and lengths of the entire guts and intestines were also measured. The livers were then used for isolation of mitochondria to measure mitochondrial respiration and antioxidant capacity (glutathione content and activities of the enzymes catalase and superoxide dismutase (SOD)) as described below.

### Isolation of hepatic mitochondria and measurement of mitochondrial respiration

Liver mitochondria were isolated according to our routine procedure [33] and were re-suspended in 3 volumes of mitochondrial respiration buffer (MRB: 10 mM Tris-HCl, 25 mM KH_2_PO_4_, 100 mM KCl, 1 mg/ml BSA, 2 μg/ml aprotonin, pH 7.3]. The mitochondrial suspensions were kept on ice and used for respiratory experiments within 4 h of isolation. We used our sequential inhibition and activation protocol [33] to measure respiration rates driven by mitochondrial complexes I-III (CI-III) in one run using Clark-type oxygen electrodes (Qubit systems, Kingston, ON). The oxygen electrodes were initially calibrated at 0 and 100% air saturation by bubbling N_2_ and air to milli-Q water, respectively, at ambient atmospheric pressure (740–760 mmHg). For each mitochondrial sample, the first measurement of respiration was done at 11 ^o^C (control, equivalent to temperature at which the feeding trial was performed) and then the temperature of the MRB in the cuvette was raised 20 ^o^C for the second measurement. Briefly cuvettes were loaded with 1.45 ml of assay temperature-equilibrated MRB and continuously stirred for homogenous distribution of O_2_ and mitochondria. Then 100 μl of mitochondrial suspension containing 2–3.5 mg protein were added followed by CI substrates (5 mM glutamate and 5 mM malate) and continuously stirred. Addition of 200 nmoles of ADP imposed maximal CI state 3 respiration rate, which transitioned to basal (state 4; proton leak) respiration rate upon depletion of the ADP. Then 0.5 μM rotenone (CI inhibitor) and 5 mM succinate (CII substrate) were introduced followed by addition of 200 nmoles of ADP to measure CII driven respiration. When CII state 3 eventually transitioned to state 4, malonate (CII inhibitor, 25 μM), 3 μM reduced decylubiquinone (CIII substrate: decylubiquinol, reduced by addition of potassium borohydride) and 200 nmoles of ADP were added to measure CIII-driven respiration. The respiratory control ratios (RCR) were calculated by dividing respective states 3 and 4 rates of respiration for each complex [34].

### Measurements of activities of catalase and SOD, and total glutathione in mitochondria

Catalase was measured using Purpald (4-amino-3-hydrazino-5-mercapto-1,2,4-triazole) based on [35] as we recently described for fish mitochondria [36]. Briefly catalase reacts with methanol in the presence of hydrogen peroxide to produce formaldehyde which upon binding to Purpald changes from colorless to purple. This color change, which is directly proportional to catalase activity, was measured by monitoring absorbance at 540 nm (SpectraMax Plus 384, Molecular Devices, LLC, Sunnyvale, CA).

For SOD, superoxide anion radical (O_2_^•–^) generated by a xanthine oxidase-hypoxanthine system was detected using water-soluble tetrazolium, WST-1 (sodium salt of 4-[3-(4iodophenyl)-2-(4-nitrophenyl)-2H-5-tetrazolio]-1,3-benzene disulfonate) according to [37] as recently described for fish mitochondria [36]. Here, WST-1 produces a water soluble dye upon reduction of the O_2_^•–^ and the rate of reduction of WST-1 is linear to xanthine oxidase activity and is inhibited by SOD. The decrease in WST-1 reduction is measured by monitoring absorbance at 440 nm (SpectraMax Plus 384) and indicates SOD activity.

Lastly, total glutathione levels were measured according to [38] as recently described for fish mitochondria [36]. The assay involves enzymatic recycling using glutathione reductase and 5–5’-dithiobis [2-nitrobenzoic acid] (DTNB) resulting in the formation of a yellow chromophore, 5-thionitrobenzoic acid (TNB), whose absorbance is measured at 412 nm (SpectraMax Plus 384). The glutathione concentrations of unknown samples were obtained by comparing their absorbance against a glutathione standard curve.

### Total plasma antioxidant capacity

The total plasma antioxidant capacity was measured using a commercial kit (Cayman Chemical, Ann Arbor, MI) according to the manufacturer’s instructions. Briefly, the ability of plasma samples to inhibit oxidation of 2,2′-azino-di-[3-ethylbenzthiazoline sulfonate]^®^ (ABTS^®^) to ABTS^®•+^ by metmyoglobin was measured by spectrophotometric monitoring of ABTS^®•+^ at 750 nm (Spectramax Plus 384). In this assay, antioxidants in the sample decrease absorbance at 750 nm to a degree proportional to their concentration.

### Biological and food utilization indexes

The following indexes were calculated:

Daily food intake (% g•g^-1^) = (food dispensed–uneaten food)/fish weight × 100.

Weight gain = final fish weight–initial fish weight.

CF: condition factor (g•cm^-1^) = weight gain/fish standard length^3^ × 100.

HSI: hepatosomatic index (% g•g^-1^) = liver weight/fish body weight × 100.

VSI: viscerosomatic index (% g•g^-1^) = viscera weight/fish body weight × 100.

SGR: specific growth rate (% bodyweight day^-1^) = (lnw_2_ − lnw_1_) × 100/T, where w_1_ and w_2_ are start and final weights respectively, ln is the natural logarithm and T the feeding days.

FCR: feed conversion ratio (g•g^-1^) = total dry feed intake/total weight gain.

PER: protein efficiency ratio (g•g^-1^) = weight gain/crude protein intake.

### Statistical analysis

The data were first tested for assumptions of normality of distribution (Kolmogorov-Smirnov) and homoscedasticity (Levene’s test) and all except the VSI data were found to conform. To reveal if the data varied among the three replicates of each experimental group, tank (replicate) effect was tested and was found not to be significant. Therefore, (i) the food consumption, growth performance and antioxidant status data were submitted to one-way analysis of variance (ANOVA) with “Seaweed supplementation” as the independent variable (ii), the mitochondrial respiration data were submitted to two-way ANOVA with “Seaweed supplementation” and “Temperature” as the independent variables and (iii), VSI data were analyzed with Kruskal-Wallis test (non-parametric one-way ANOVA). Statistica 13.3 (TIBCO Software, Palo Alto, CA, USA) was used for all the analyses. Note that percent data were arcsine-transformed before the statistical analysis. The least significant difference (LSD) test was used for post hoc comparison of means when the ANOVA main effects were significant. The level of significance for all of the analyses was set at p < 0.05.

## Results

### Seaweed and food composition

Analysis of the seaweed showed that it contained 12.37% crude protein and non-detectable levels of fat on dry matter basis (Table 1). Additionally, relative to the control commercial salmonid feed, the seaweed contained lower levels of key minerals (phosphorus, copper and zinc) and higher levels of neutral detergent fiber (NDF), ash and NaCl. Addition of the seaweed to commercial salmonid feed caused slight changes in the proportion of other dietary ingredients (Tables 1 and 2). Notably, the crude protein (dry matter basis, Table 1) decreased from 57.6% (control) to 52.7 (10% seaweed) and from 54.7 to 48.9% (as fed basis, Table 2) in control and 10% seaweed-supplemented food, respectively. To put these values into nutritional requirement context, levels of crude protein in feeds for various age classes of Atlantic salmon range from 42–50%, with 45% for juveniles [39]. Similarly, the slightly reduced levels of phosphorus, copper and zinc in the seaweed-supplemented diets more than met the respective minimum nutritional requirements for various life stages/size classes of Atlantic salmon [39]. Based on these findings, it is likely that higher rates of inclusion (>10%) of this seaweed might decrease levels of key ingredients to levels below their minimum requirements.

**Table 1:** Food and seaweed composition (dry matter basis).

Analyte	Food				
	Control	SW-3%	SW-6%	SW-10%	SW-100%
Crude protein (%)	57.55	55.98	55.24	52.72	12.37
NDF (%)	12.27	15.08	12.04	18.27	42.13
Calcium (%)	3.09	3.06	3.04	3.08	2.77
Phosphorus (%)	1.96	2.04	1.88	1.76	0.14
Magnesium (%)	0.23	0.25	0.26	0.29	0.74
Potassium (%)	0.90	0.93	0.97	1.04	1.86
Copper (ppm)	11.98	10.20	9.73	10.81	<3.00
Zinc (ppm)	169.4	150.3	136.8	136.2	15.03
Sodium (%)	0.86	0.92	0.99	1.07	2.45
NaCl (as Na) (%)	2.18	2.35	2.52	2.72	6.21
Fat (%)	12.36	12.08	11.71	11.16	<1.00
Ash (%)	12.84	13.52	14.35	15.16	39.70

SW: seaweed; NDF: neutral detergent fiber.

**Table 2:** Food and seaweed composition (as fed basis).

Analyte	Food				
	Control	SW-3%	SW-6%	SW-10%	SW-100%
Dry matter (%)	95.1	94.55	94.29	92.75	85.44
Crude protein (%)	54.73	52.93	52.08	48.9	10.57
NDF (%)	11.67	14.25	11.35	16.95	36
Calcium (%)	2.94	2.89	2.86	2.86	2.36
Phosphorus (%)	1.87	1.93	1.77	1.63	0.12
Magnesium (%)	0.22	0.24	0.25	0.27	0.63
Potassium (%)	0.86	0.88	0.92	0.97	1.59
Copper (ppm)	11.39	9.65	9.17	10.02	<3.00
Zinc (ppm)	161.1	142.1	128.9	126.3	12.84
Sodium (%)	0.82	0.87	0.94	0.99	2.09
NaCl (as Na) %)	2.07	2.22	2.38	2.52	5.31
Fat (%)	11.76	11.43	11.04	10.35	<1.00
Ash (%)	12.21	12.78	13.53	14.06	33.92

SW: seaweed; NDF: neutral detergent fiber.

### Seaweed supplementation increases food consumption and performance

**Fig 1:**
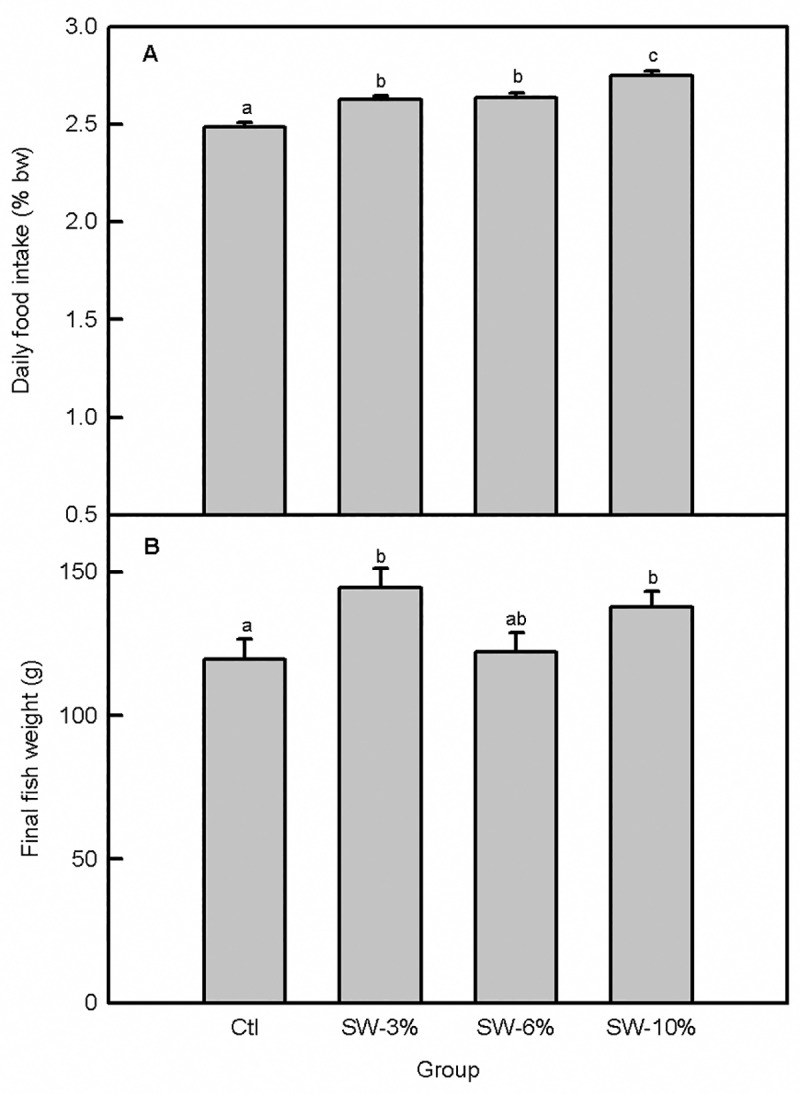
Brown seaweed supplementation increases food intake and body weight in Atlantic salmon smolts. (A) Daily food intake. (B) Final fish weight. Ctl: control (0% seaweed), SW-3%: 3% seaweed, SW-6%: 6% seaweed, SW-10%: 10% seaweed. Bars with different letters are significantly different (one-way ANOVA, LSD test, p < 0.05).

**Fig 2:**
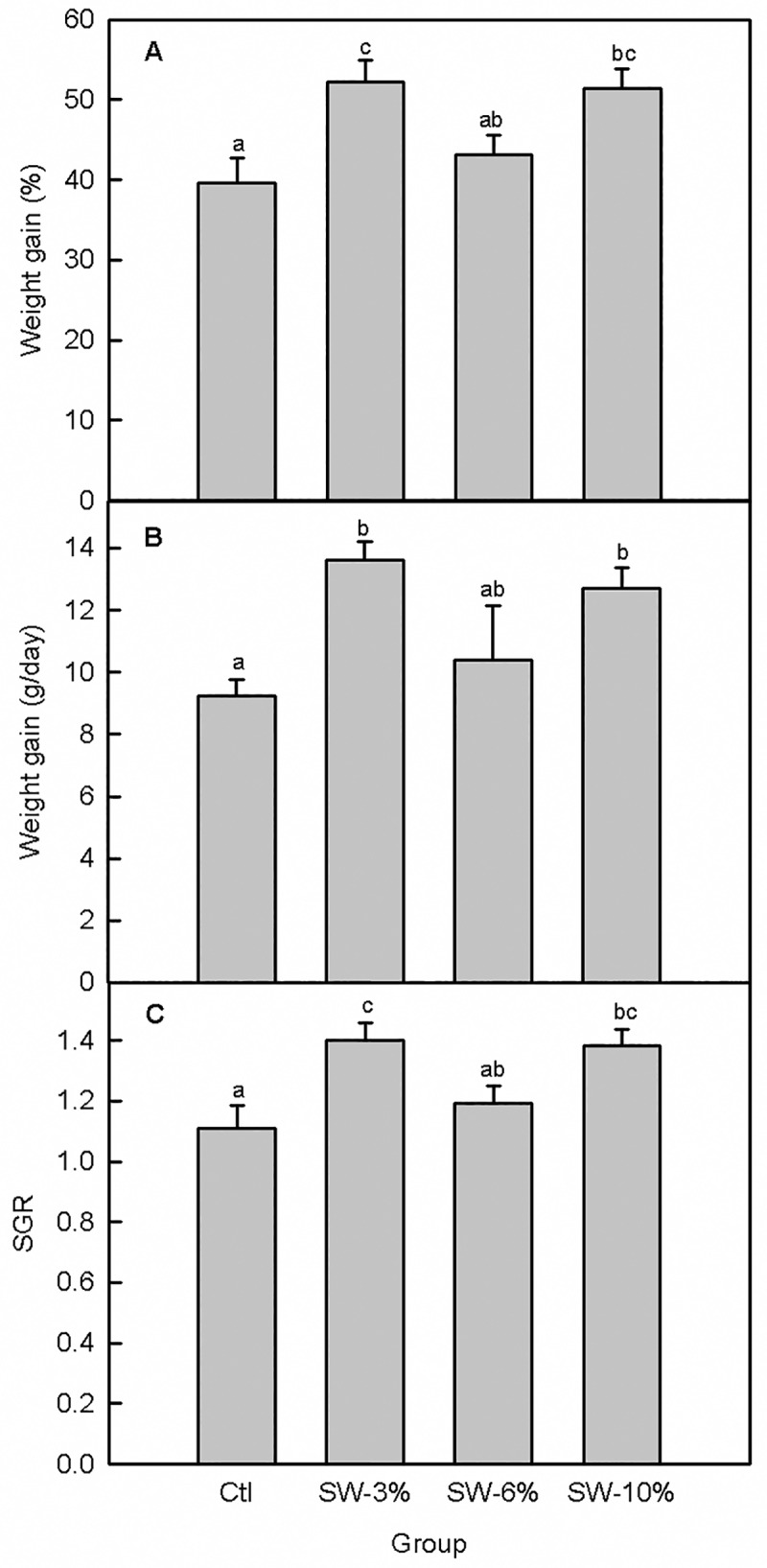
Effect of brown seaweed supplementation on growth performance indices of Atlantic salmon smolts. (A) % weight gain. (B) Daily weight gain. (C) SGR. Ctl: control (0% seaweed), SW-3%: 3% seaweed, SW-6%: 6% seaweed, SW-10%: 10% seaweed. Bars with different letters are significantly different (one-way ANOVA, LSD test, p < 0.05).

**Fig 3:**
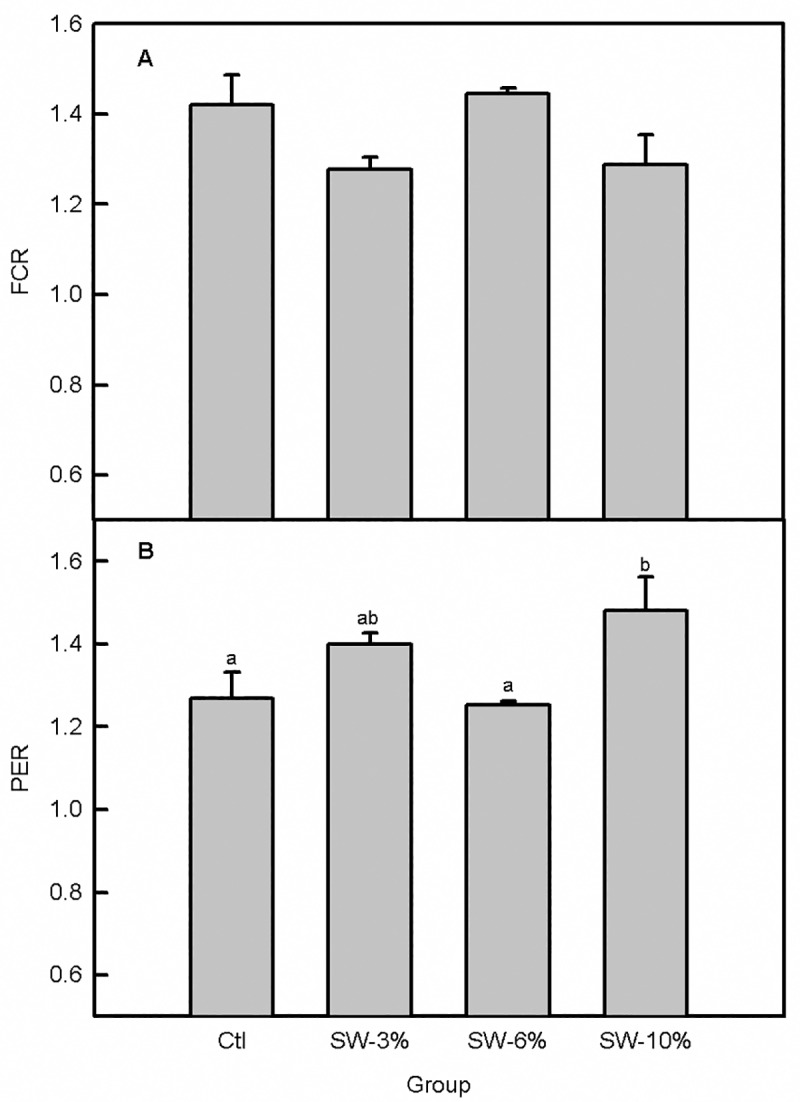
Supplementation with brown seaweed improves PER but not FCR in Atlantic salmon smolts. (A) FCR. (B) PER. Ctl: control (0% seaweed), SW-3%: 3% seaweed, SW-6%: 6% seaweed, SW-10%: 10% seaweed. Bars with different letters are significantly different (one-way ANOVA, LSD test, p < 0.05).

There were no mortalities during the 30-day feeding trial; all of the fish in each of the 12 tanks survived and no malformations or other adverse effects were observed. The fish readily accepted the experimental food and the overall effect of seaweed supplementation on food intake (Fig 1A) was significant (F_3,356_ = 22.6, p < 0.0001). Specifically, fish maintained on the seaweed-supplemented diets consumed significantly more food than the control. Consistent with the pattern of food consumption (Fig 1A), fish weight per tank varied with seaweed supplementation (F_3,92_ = 3.56, p = 0.02), with fish maintained on food supplemented with 3 and 10% seaweed having significantly higher final body weights than the control (Fig 1B). The higher final fish weights relative to the control for the 3 and 10% seaweed-supplemented fish were associated with higher % weight gain (F_3,8_ = 5.36, p = 0.03), higher daily weight gain (F_3,92_ = 4.06, p = 0.05), and higher SGR (F_3,92_ = 5.35, p = 0.03) (Fig 2A–2C). Although seaweed supplementation overall did not significantly alter the FCR (F_3,8_ = 3.14, p = 0.08) (Fig 3A), the 3% supplemented group had better FCR than the 6% supplemented group based on an independent Student’s t-test (comparison not shown). Moreover, PER was significantly altered by seaweed supplementation (F_3,8_ = 4.23, p = 0.04) (Fig 3B) and was higher in the 3 and 10% groups relative to the 6% group.

**Fig 4:**
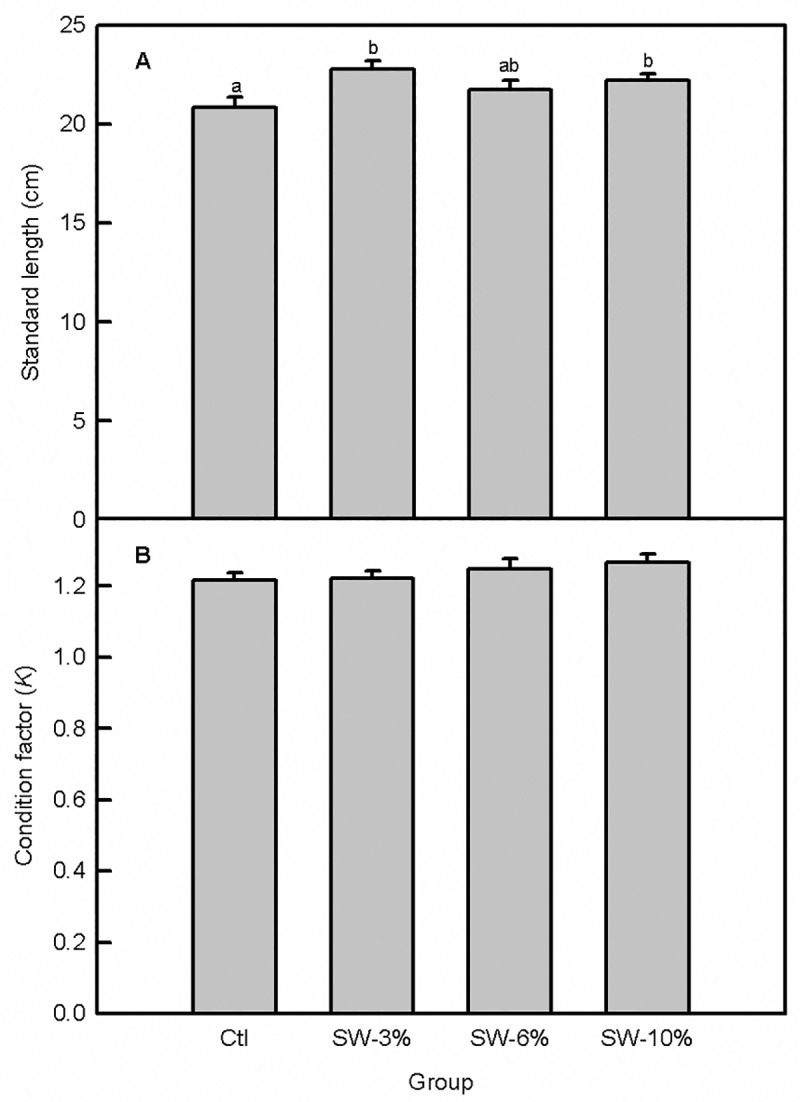
Effect of brown seaweed supplementation on body length and *K* of Atlantic salmon smolts. (A) Standard length. (B) Condition factor (*K*). Ctl: control (0% seaweed), SW-3%: 3% seaweed, SW-6%: 6% seaweed, SW-10%: 10% seaweed. Bars with different letters are significantly different (one-way ANOVA, LSD test, p < 0.05).

Seaweed supplementation significantly altered fish standard length (F_3,92_ = 3.4, p = 0.02) in which fish that were fed 3 and 10% seaweed-supplemented food were longer than the control at the end of the trial (Fig 4A). However, *K* computed from the final weights and standard lengths remained unchanged (F_3,92_ = 0.91, p = 0.44; Fig 4B). Measurement of other morphometric indices revealed that seaweed supplementation did not alter body depth, HSI and VSI, but it increased weights and lengths of the entire guts and intestines for the 3 and 10% supplementation levels relative to the control (Table 3).

**Table 3:** Effect of brown seaweed supplementation on morphometric indices of Atlantic salmon smolts.

Index	Control	SW-3%	SW-6%	SW-10%	F Statistic (p value)
Body depth (cm)	4.43±0.09	4.58±0.08	4.39±0.10	4.51±0.06	1.03 (0.39)
HSI	0.93±0.03	0.90±0.03	0.85±0.03	0.89±0.02	1.60 (0.22)
VSI	9.78±0.61	9.05±0.33	9.63±0.61	9.62±0.46	χ^2^_(3)_ = 0.33 (0.95)*
Gut weight (g)	7.30±0.40^a^	8.66±0.43^b^	7.65±0.46^ab^	8.71±035^b^	2.99 **(0.04)**
Gut length (cm)	17.5±0.63^a^	19.9±0.53^b^	19.0±0.69^ab^	20.0±0.42^b^	4.10 **(<0.01)**
Intestine weight (g)	6.09±0.34^a^	7.19±0.36^c^	6.16±0.39^ab^	7.09±0.34^bc^	3.16 **(0.03)**
Intestine length (cm)	14.0±0.54^a^	15.9±0.49^b^	15.0±0.55^ab^	15.9±0.40^b^	3.05 **(0.03)**

HSI: hepatosomatic index; VSI: viscerosomatic index; SW: seaweed. Values in a row with different letters are significantly different (one-way ANOVA, LSD test, p < 0.05). The F statistics degrees of freedom were 3 for seaweed supplementation groups and 92 for sample size except for HSI which were 3 and 20, respectively. The bolded p values are significant. Asterisk (*) indicates Kruskal-Wallis test was used for analysis.

### Seaweed supplementation increases total plasma and mitochondrial antioxidant capacities

**Fig 5:**
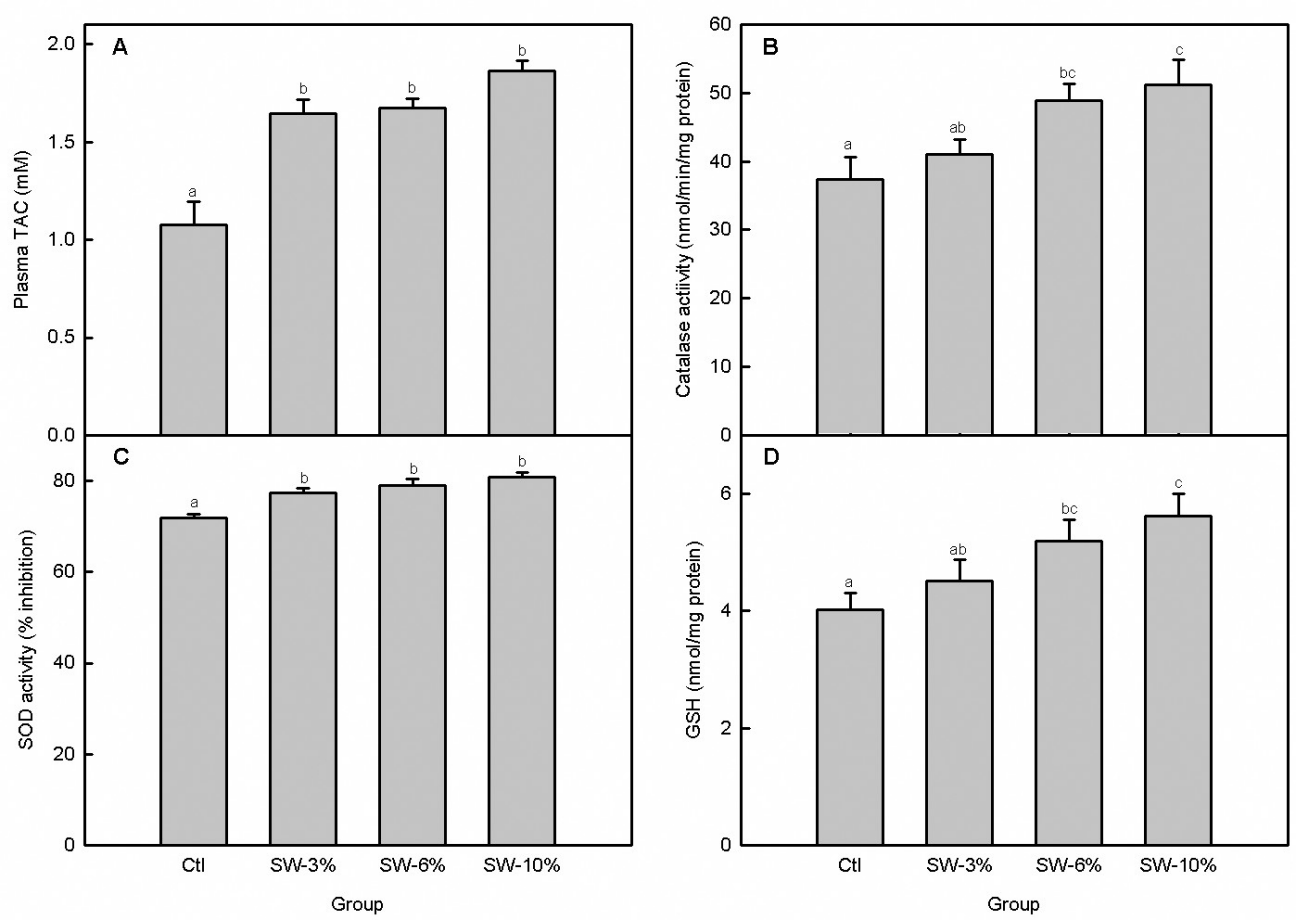
Effect of brown seaweed supplementation on antioxidant status in Atlantic salmon smolts. (A) Plasma total antioxidant capacity, TAC. (B) Catalase activity. (C) Total superoxide dismutase (SOD) activity. (D) Total glutathione. Ctl: control (0% seaweed), SW-3%: 3% seaweed, SW-6%: 6% seaweed, SW-10%: 10% seaweed. Bars with different letters are significantly different (one-way ANOVA, LSD test p < 0.05).

The overall effect of dietary seaweed supplementation on plasma total antioxidant capacity, TAC (Fig 5A) was highly significant (F_3,20_ = 18.7, p < 0.0001). Specifically, all the three levels of seaweed supplementation significantly increased the plasma TAC relative to the control but there were no differences among the seaweed-supplemented groups.

To assess mitochondrial antioxidant capacity, we measured activities of catalase and SOD, and levels of glutathione. We found that seaweed-supplementation concentration-dependently increased the activities of catalase (F_3,20_ = 4.85, p = 0.01; Fig 5B) and total SOD (F_3,20_ = 7.6, p = 0.001; Fig 5C) as well as the level of total glutathione (F_3,20_ = 4.03, p = 0.02; Fig 5D). In particular, catalase activity and total glutathione content were significantly higher than the control for the 6 and 10% seaweed-supplementation while SOD activity was higher than the control for all of the three levels of seaweed-supplementation.

### Seaweed supplementation reduces temperature-responsiveness of mitochondrial respiration

**Fig 6:**
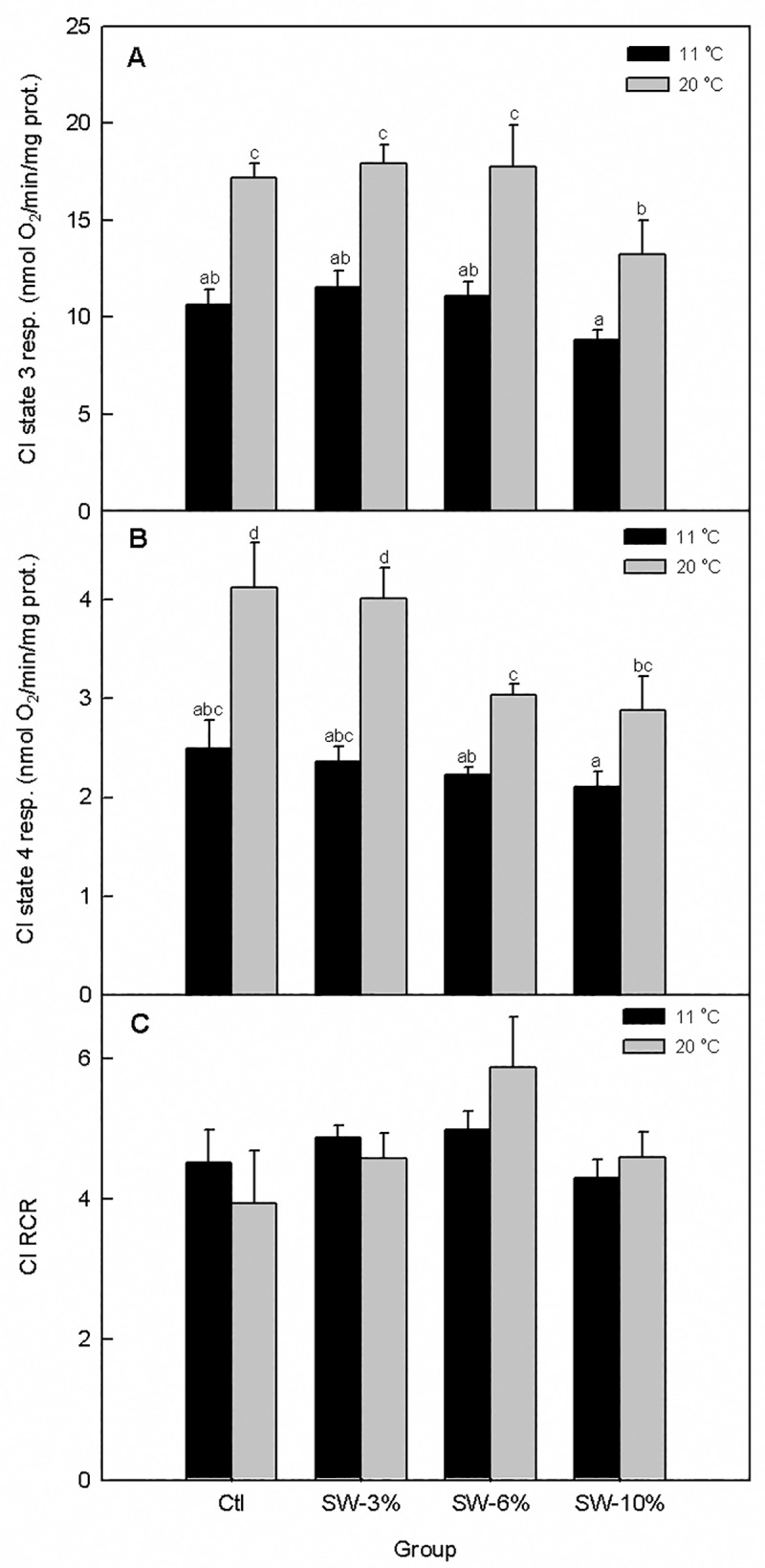
Effect of brown seaweed supplementation and temperature on mitochondrial complex I-supported respiration in Atlantic salmon smolts. (A) State 3 respiration. (B) State 4 respiration. (C) RCR. Ctl: control (0% seaweed), SW-3%: 3% seaweed, SW-6%: 6% seaweed, SW-10%: 10% seaweed. Bars with different letters are significantly different (ANOVA, p < 0.05). Bars with different letters are significantly different (two-way ANOVA, LSD test, p < 0.05).

We then assessed the effect of seaweed supplementation on mitochondrial respiration and its responsiveness to acute temperature rise (11 → 20 ^o^C) *in vitro* for CI-III-supported respiration rates. We found that temperature (F_1,40_ = 50.4, p < 0.0001) and seaweed supplementation (F_3,40_ = 4.0, p = 0.01) significantly altered CI-supported state 3 mitochondrial respiration albeit without a significant interaction (F_3,40_ = 0.42, p = 0.74) (Fig 6A). Notably, CI-supported state 3 respiration rate exhibited a smaller response to temperature elevation in fish fed 10% seaweed relative to the control. Similarly, temperature (F_1,40_ = 42.4, p < 0.0001) and seaweed supplementation (F_3,40_ = 4.6, p = 0.007) significantly altered CI state 4 mitochondrial respiration (proton leak) without a significant interaction (F_3,40_ = 1.69, p = 0.19) (Fig 6B). Here, CI state 4 respiration rates exhibited smaller responses to temperature elevation for the 6 and 10% levels of seaweed supplementation. In contrast to the clear changes in state 3 and 4 respiration rates, temperature (F_1,40_ = 0.06, p = 0.81) and seaweed supplementation (F_3,40_ = 2.55, p = 0.07) did not alter CI RCR (Fig 6C) nor was the interaction term significant (F_3,40_ = 1.29, p = 0.41).

**Fig 7:**
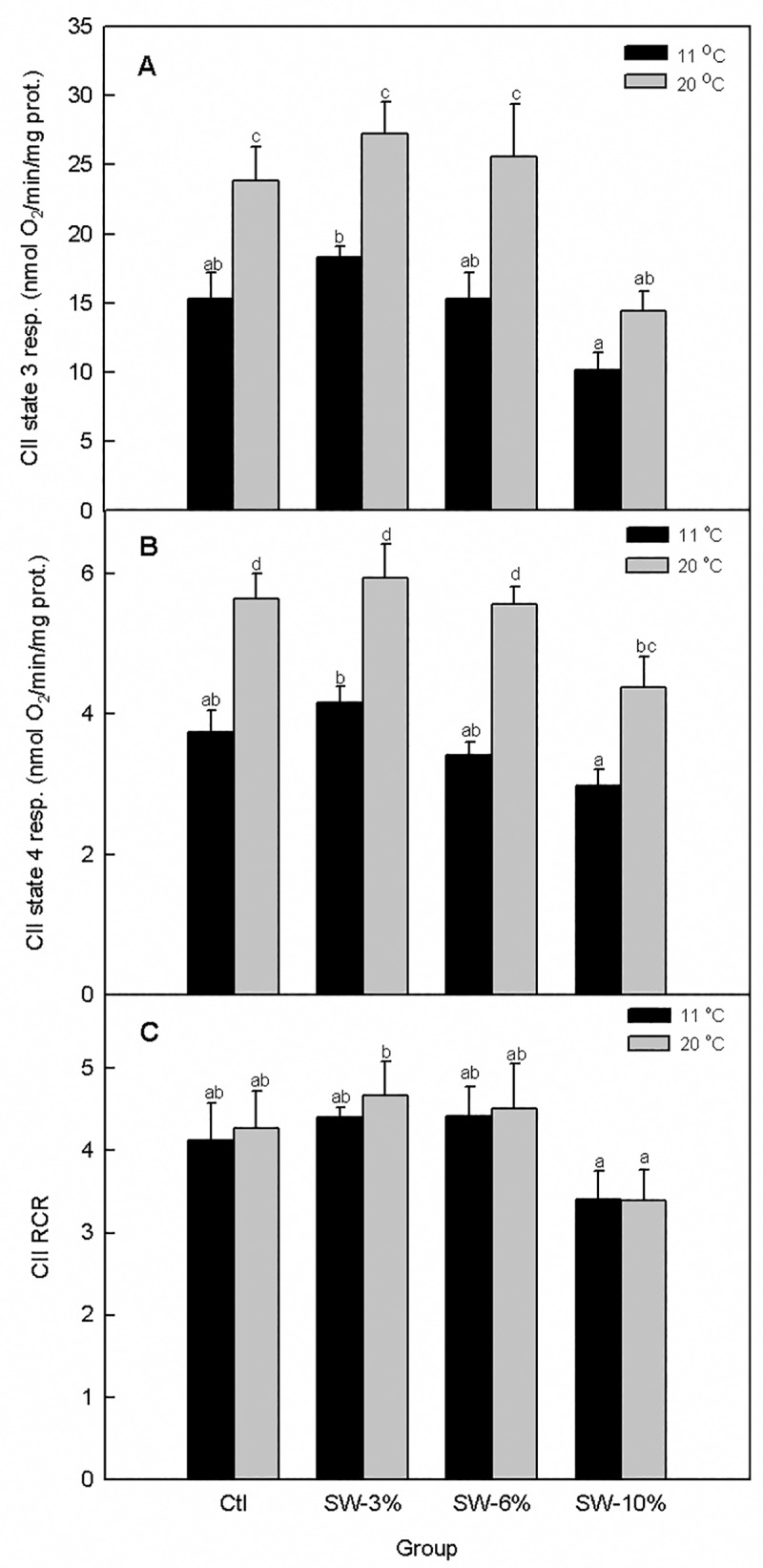
Effect of brown seaweed supplementation and temperature on mitochondrial complex II-supported respiration in Atlantic salmon smolts. (A) State 3 respiration. (B) State 4 respiration. (C) RCR. Ctl: control (0% seaweed), SW-3%: 3% seaweed, SW-6%: 6% seaweed, SW-10%: 10% seaweed. Bars with different letters are significantly different (ANOVA, p < 0.05). Bars with different letters are significantly different (two-way ANOVA, LSD test, p < 0.05).

Temperature (F_1,40_ = 27.9, p < 0.0001) and seaweed supplementation (F_3,40_ = 8.81, p = 0.0001) significantly altered CII state 3 mitochondrial respiration without a significant interaction (F_3,40_ = 0.73, p = 0.54) (Fig 7A). Notably, dietary supplementation with 10% seaweed decreased temperature responsiveness of CII state 3 respiration relative to the control. Surprisingly, CII state 3 respiration rates measured at 11 and 20 ^o^C were not statistically different from each other. We additionally found that temperature (F_1,40_ = 62.1, p < 0.0001) and seaweed supplementation (F_3,40_ = 6.43, p = 0.001) significantly altered CII state 4 respiration without a significant interaction (F_3,40_ = 1.18, p = 0.35) (Fig 7B). Here, the temperature-imposed increase in state 4 respiration rate was lower for the 10% seaweed supplementation relative to the control. Lastly, CII RCR (Fig 7C) was significantly altered by seaweed supplementation (F_3,40_ = 3.43, p = 0.03) but not temperature (F_1,40_ = 0.19, p = 0.67), and the interaction of the two factors was not significant (F_3,40_ = 0.04, p = 0.99).

**Fig 8:**
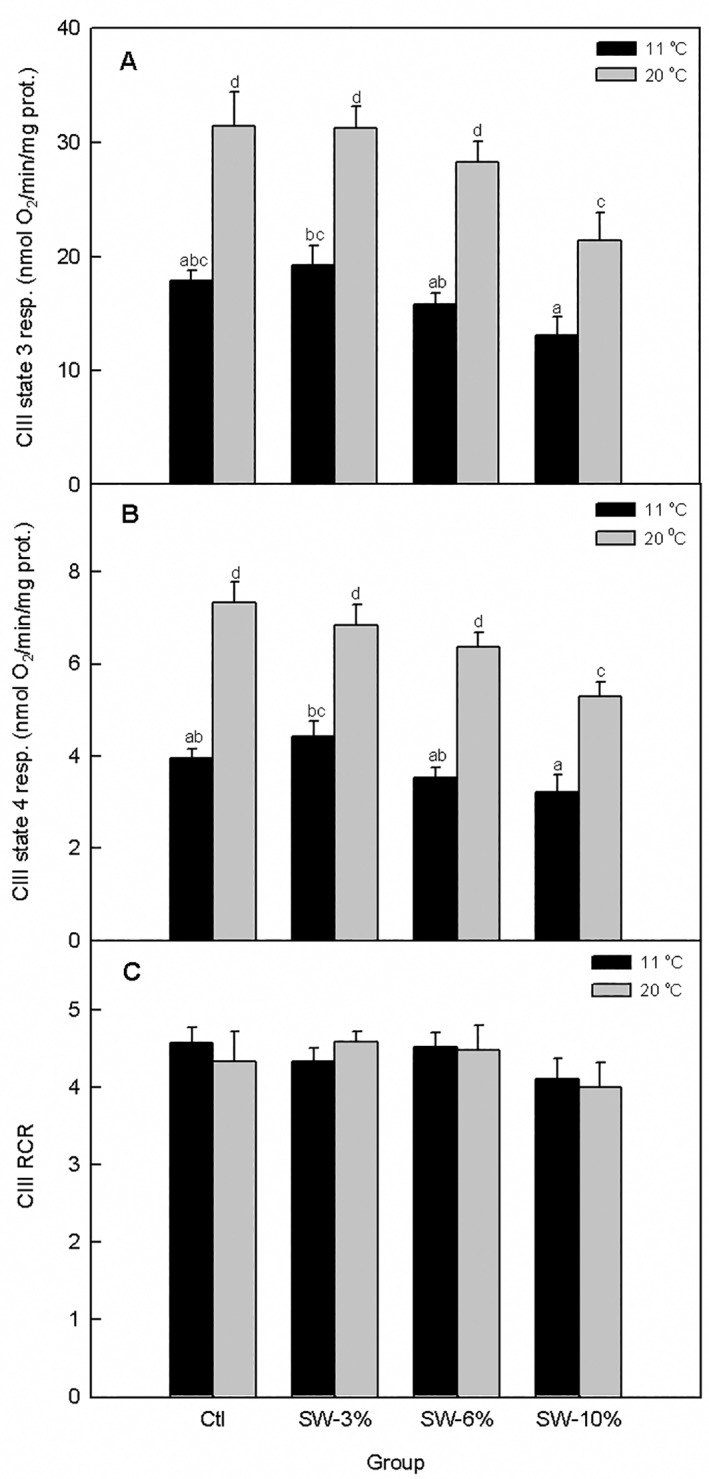
Effect of brown seaweed supplementation and temperature on mitochondrial complex III-supported respiration in Atlantic salmon smolts. (A) State 3 respiration. (B). State 4 respiration. (C) RCR. Ctl: control (0% seaweed), SW-3%: 3% seaweed, SW-6%: 6% seaweed, SW-10%: 10% seaweed. Bars with different letters are significantly different (ANOVA, p < 0.05). Bars with different letters are significantly different (two-way ANOVA, LSD test, p < 0.05).

Temperature (F_1,40_ = 73.6, p < 0.0001) and seaweed supplementation (F_3,40_ = 7.31, p = 0.0005) significantly altered CIII state 3 mitochondrial respiration without a significant interaction (F_3,40_ = 0.70, p = 0.55) (Fig 8A). Importantly, CIII-supported state 3 respiration rate showed a smaller response to temperature elevation in fish fed 10% seaweed relative to the control. As well, CIII state 4 respiration rate was significantly altered by temperature (F_1,40_ = 119, p < 0.0001) and seaweed supplementation (F_3,40_ = 7.3, p = 0.0005) without a significant interaction of the two factors (F_3,40_ = 1.33, p = 0.28) (Fig 8B). Similar to CI and II, CIII state 4 respiration rate exhibited smaller response to temperature elevation for the 10% levels of seaweed supplementation. However, CIII RCR (Fig 8C) was not significantly altered by temperature (F_1,40_ = 0.03, p = 0.86) and seaweed supplementation (F_3,40_ = 1.26, p = 0.3) nor was the interaction of the two factors significant (F_3,40_ = 0.30, p = 0.83).

## Discussion

Although seaweeds are considered natural forage for fish, they remain underutilized as an ingredient in aquafeeds. We tested the potential use of seaweed meal derived from brown seaweed of the genus Laminaria (kelp) as an additive to commercial salmonid food using Atlantic salmon smolts. Because of their large size and ease of harvesting, brown seaweeds are generally more amenable to exploitation for animal nutrition than other types of macroalgae. However, brown seaweeds have low nutritional value, containing only 3–15% crude protein compared with green and red seaweeds that may contain up to 26 and 47% crude protein, respectively [16]. The batch of seaweed meal (AquaArom) we used was tested and was found to contain 12.37% protein on a dry matter basis which is within the range for brown seaweeds. Despite their low protein content, brown seaweeds remain popular as potential animal feed additives because they are rich in bioactive compounds [23]. Indeed, brown seaweeds constitute the majority of seaweed used in terrestrial animal nutrition [12,23] but they are the least investigated for application in aquafeeds relative to other seaweed classes [4]. In our study, Atlantic salmon smolts readily accepted food mixed with 3–10% brown seaweed. Interestingly, smolts maintained on seaweed-supplemented feed consumed more food than the control. This contrasts the typical finding that inclusion of vegetable protein, particularly in high amounts, in fish diets is associated with reduced palatability and food intake, resulting in decreased growth performance [3,7,40,41]. Regardless, increased intake of seaweed-supplemented fish food has also been previously reported and attributed to the presence in seaweeds of compounds such as dimethyl sulfonyl propionate, dimethyl-beta-propionthein, and amino acids that attract fish to food thus enhancing consumption [12,42,43]. An alternative explanation for the increased food intake could be that increased fiber content due to the added seaweed would reduce available dietary energy resulting in increased food intake as a mechanism to compensate for the energy shortfalls [44]. Moreover, the addition of seaweed to aquafeeds can improve the texture, integrity, and water stability of pellets resulting in higher food consumption [45,46].

The common theme emergent from studies assessing the utility of seaweeds as a nutritional ingredient in aquafeeds is that the biological responses depend on the seaweed and fish species, and the level of inclusion [14,20, 27,28, 40]. Favorable effects of seaweed supplementation in aquafeeds typically occur at inclusion levels of up to 10% and include improved survival, improved growth rate and feed utilization efficiency, and increased assimilation of dietary nutrients [14,28]. In our study, supplementation with 3–10% brown seaweed did not affect survival during the 30-day feeding trial. Importantly, fish fed 3 and 10% seaweed-supplemented food exhibited higher daily weight gains, SGR and both final weight and total length than the control, while fish fed 6% seaweed maintained growth comparable to the control. Moreover, seaweed supplementation increased PER and imposed a strong tendency to improve FCR (F statistic p value = 0.08). Two previous studies that assessed the effect of diets supplemented with the brown seaweed, *Laminaria digitata*, in salmonids found that in Atlantic salmon, food intake and growth performance were not affected [47] while in rainbow trout the final body weight increased, FCR decreased and food intake remained unchanged [48]. More generally, the potential utility of other types of seaweeds as supplements or protein sources for salmonids has been investigated [19,26,49,50]. It was found that the inclusion of up to 10% *Porphyra dioica* (red seaweed) did not impair growth performance in rainbow trout (*Oncorhynchus mykiss*) but 15% inclusion reduced the final weight of the fish [49]. The macroalgae product Verdemin (derived from the green seaweed, *Ulva ohnoi*) at inclusion levels of 2.5 and 5.0% in juvenile Atlantic salmon diets did not alter feed efficiency and growth performance [19]. Additionally, feeding rainbow trout diets supplemented with *Gracilaria pygmaea* improved growth performance at 6% inclusion level; however, growth was impaired at 12% inclusion [50]). Further, it was found that the inclusion of 5% *Gracilaria vermiculophylla* in rainbow trout diet did not negatively impact growth performance but 10% inclusion reduced the final weight, body length, daily growth index and PER [26]. For the Atlantic salmon, 5–15% supplementation of *Palmaria palmata* in formulated diets did not affect growth performance [51]. Overall, our findings together with these earlier studies indicate that supplementation of aquafeed with seaweeds at appropriate levels can enhance or not alter growth performance in salmonids.

The enhancement of growth by seaweeds has been attributed to seaweed vitamin and mineral content [45] and/or increased lipid mobilization and assimilation of dietary nutrients [52–54]. In our study, it is unlikely that phosphorus, copper and zinc contributed to the growth-stimulatory effects because the seaweed we tested contained low levels of these minerals. It has also been postulated that prebiotic activity of seaweed components (e.g., polysaccharides and oligosaccharide) stimulates growth of beneficial bacteria thereby improving digestion and subsequently growth [55]. While we did not investigate the mechanisms or causes of the enhanced growth performance, we found that the digestive tracts were heavier and longer in fish fed seaweed-supplemented diets suggesting a larger surface area for digestion and absorption of nutrients. Longer digestive tracts would increase the retention time of food allowing more time for digestion of seaweed-supplemented diets. Surprisingly, the highest seaweed inclusion level (10%) we tested resulted in better growth performance despite a 5%-point lower protein content relative to the control (Tables 1 and 2). Regardless, the protein contents for all the seaweed-supplemented diets were within the optimal range for Atlantic salmon nutrition [39]. Overall, while it appears that up to 10% inclusion of AquaArom stimulates growth in Atlantic salmon smolts, a longer feeding trial would be necessary for a more definitive conclusion on growth performance. Furthermore, future studies should investigate the mechanisms and components underlying the growth-enhancing effects of AquaArom.

The variant growth response commonly reported among different fish when fed seaweed-supplemented diets can, in part, be explained by fish natural feeding strategies and gut morphology which dictate the ability (e.g., presence of necessary digestive enzymes) of the fish to digest and absorb nutrients in the seaweed [56]. Generally, herbivorous (and omnivorous) fish, e.g., the common carp (*Cyprinus carpio*) and Nile tilapia (*Oreochromis niloticus*) have high amylase activity which facilitates digestion of seaweeds [19,57,58]. In contrast, carnivorous fish like Atlantic salmon and trout have limited ability to hydrolyse complex polysaccharides present in seaweeds because of the absence or low levels of requisite enzymes [58,59]. It is also possible that growth impairment results from high levels of anti-nutrient compounds present in seaweeds that inhibit nutrient absorption in the digestive system [3,19,60]. For example, brown algae species contain pholorotannins that are known to inhibit fish digestive enzymes [61]. Another potential cause of growth impairment is that polysaccharides present in seaweeds may impose rapid passage of food through the digestive tract thus increasing the feed intake but reducing nutrient absorption [60,62,63]. Apparently for our study, the impact of these growth-limiting mechanisms was not significant to alter growth performance in Atlantic salmon smolts fed diets supplemented with 3–10% brown seaweed.

Representative species of the three seaweed phyla (Chlorophyta, Rhodophyta and Phaeophyta) are known to contain a wide array of bioactive compounds making seaweeds an important potential source of nutraceuticals for animal feed fortification [64]. These bioactive compounds (secondary metabolites) are believed to be synthesized as adaptive responses to multiple stressors that prevail in seaweed habitats [4,12,65]. In brown seaweeds, a representative of which we tested in our study, these compounds include polyphenols, phenolic compounds and sulphated polysaccharides [66–68]. Indeed, red and green seaweeds also contain polysaccharides and/or phenols/flavonoids [50,69–71]. These compounds have multiple properties including antimicrobial, immunostimulant, anti-viral, and antioxidant activities [72–74]) and can modulate numerous biological processes leading to improved physiological and health status. Here, we assessed the effect of feeding Atlantic salmon smolts seaweed-supplemented diets on antioxidant status in plasma and mitochondria. All of the levels of seaweed supplementation we tested increased plasma total antioxidant capacity relative to the control. Furthermore, seaweed-supplementation concentration-dependently increased the activities of mitochondrial antioxidant enzymes (catalase and SOD), and the levels of total glutathione. Our findings are consistent with previous reports that dietary seaweed supplementation modulates antioxidant status and oxidative stress in farmed animals. For example, inclusion of *Gracilaria pygmaea* in the diet of rainbow trout reduced SOD and glutathione peroxidase activities and lipid peroxidation in liver indicative of reduced need to scavenge reactive oxygen species (ROS) [50]. Glutathione peroxidase and glutathione s-transferase activities and lipid peroxidation were increased following supplementation of the European seabass (*Dicentrarchus labrax*) food with seaweeds of *Gracilaria* sp. [27,28]. In ruminants, dietary supplementation with the brown seaweed (*Ascophyllum nodosum*) meal or its extract increased serum antioxidant status, reduced lipid peroxidation, and increased activities of SOD and glutathione peroxidase [75–78]. Importantly, direct free radical scavenging activity of extracts from brown (and red) seaweed has been demonstrated *in vitro* [79–82]. Thus our study, together with these earlier reports, suggests that dietary brown seaweed supplementation enhances antioxidant capacity directly by increasing levels of antioxidant compounds in circulation/tissues and indirectly by modulating activities of antioxidant defense systems.

In aquaculture settings, fish experience many types of stress, both physical and environmental. Heat stress is particularly common in summer and has been shown to impair performance of terrestrial farm animals [83,84]. Importantly, the effect of heat stress in animal production is predicted to worsen due to the global warming phenomenon [85]. Clearly, a need exists to develop nutritional interventions to alleviate the adverse effects of heat in farmed animals. Therefore, to assess the role of seaweed supplementation on heat stress response, we measured respiration in mitochondria obtained from livers of Atlantic salmon smolts fed diets containing 0% (control) and 3–10% brown seaweed following acute temperature rise *in vitro*. Because high temperature increases energy metabolism and mitochondria are the main sites of cellular energy conversion, we hypothesized that mitochondria would be an ideal model to test effect of dietary seaweed supplementation on thermal stress. We found that liver mitochondrial respiration supported by CI-III in fish fed seaweed-supplemented diets (6 and 10%) exhibited smaller increases when subjected to acute temperature rise than those from fish maintained on control diet. However, the RCR was not altered indicating that the relative changes in states 3 and 4 respiration were similar, and that the temperature challenge we tested did not alter mitochondrial coupling efficiency. While effect of seaweed supplementation on responses to acute temperature stress has not been tested in fish, brown seaweed (*A*. *nodosum*) meal protected lambs and kids against heat-induced oxidative stress [77,86]. Heat stress may modulate oxidative stress by altering production of ROS and/or activities of antioxidant defence systems [86–88]. Indeed, the involvement of ROS (oxidative stress) in heat stress pathophysiology is supported by the finding that supplementation of sheep diets with the antioxidants vitamin E and Se reduced heat-induced oxidative stress [89]. More pertinently, it has been shown by direct measurement in isolated mitochondria that an increase in temperature increases ROS emission [90–93]). While we did not directly measure ROS emission, the elevation of state 4 rate of respiration during acute temperature rise (Figs 6–8) is indicative of elevated mitochondrial membrane potential which favors mitochondrial ROS production [94–95]. Our finding that seaweed supplementation (6 or 10%) resulted in lower increases in state 4 respiration rate following acute temperature rise suggests that ROS production would be reduced but this remains to be directly tested. Moreover, the role of seaweed supplementation in aquafeeds in alleviating effects of stressors is not limited to temperatures stress. For example, higher survival rate was reported in sea bream fed diets supplemented with *Gacilaria* sp. relative to the control following exposure to hypoxia [96]. Interestingly, the increased survival was associated with decreased lipid peroxidation and altered gene expression of antioxidant enzymes suggesting protection against oxidative stress was the underlying mechanism.

## Conclusions

Overall, our study shows that the addition of AquaArom to commercial salmonid food increases food intake and enhances growth performance, improves plasma antioxidant capacity and alleviates the effect of temperature rise on mitochondrial respiration. The slight decline in crude protein and minerals resulting from the addition of up to 10% AquaArom to aquafeed appear to have no adverse consequences on Atlantic salmon smolts. Thus, mixing of brown seaweed meal with commercial aquafeeds (and potentially feeds for other farm animals) could offer a cost-effective way of harnessing the beneficial effects of seaweeds in animal production.

## Supporting information

S1 Table Sources and descriptions of methods used for analysis of the composition of experimental diets and seaweed (AquaArom). (DOCX)
