# Supplementary material for: Brown seaweed (AquaArom) supplementation increases food intake and improves growth, antioxidant status and resistance to temperature stress in Atlantic salmon, Salmo salar
Source: PLoS One. 2019 Jul 15;14(7):e0219792. doi: 10.1371/journal.pone.0219792 (PMC6629153; doi:10.1371/journal.pone.0219792)
Supplement: S1 Table — (DOCX) [file pone.0219792.s001.docx]

**S1 Table**

| **Analyte** | **Reference Method** | **Brief Description and Source** |
| --- | --- | --- |
| Crude protein | Modified Protein (Crude) in Animal Feed: Combustion Method (990.03) - Official Methods of Analysis of AOAC International. | Nitrogen is released from samples by combustion at high temperature in pure oxygen and measured by thermal conductivity, and converted to equivalent protein by appropriate numerical factor.  <https://www.aoac.org/aoac_prod_imis/AOAC/Publications/Official_Methods_of_Analysis/AOAC_Member/Pubs/OMA/AOAC_Official_Methods_of_Analysis.aspx> |
| Crude fat | Reference: ANKOM Technology Method 2, 01-30-09 | Samples are heated under pressure in petroleum ether to extract lipids (primarily triacylglycerides with small amounts of other lipids). The percent crude fat is then determined from sample weights before and after the extraction.  <https://www.ankom.com/sites/default/files/document-files/Crude_Fat_Abstract.pdf> |
| Ash | Ash of Animal Feeds (942.05) - Official Methods of Analysis of AOAC International | A ground sample is ignited in a furnace at 600 °C to oxidize all organic matter. Ash is determined by weighing the resulting inorganic residue.  <https://www.aoac.org/aoac_prod_imis/AOAC/Publications/Official_Methods_of_Analysis/AOAC_Member/Pubs/OMA/AOAC_Official_Methods_of_Analysis.aspx> |
| Minerals | Minerals in Animal Feed and Pet Food, Atomic absorption spectrophotometric method (968.08) - Official Method of Analysis of AOAC International | A dried ground sample is ignited in a furnace at 550 ^o^C ± 10 ^o^C to oxidize all organic matter. The remaining ash is dissolved in hydrochloric acid and analyzed using Inductively Coupled Argon Plasma Optical Emission Spectrometer (ICP-OES).  <https://www.aoac.org/aoac_prod_imis/AOAC/Publications/Official_Methods_of_Analysis/AOAC_Member/Pubs/OMA/AOAC_Official_Methods_of_Analysis.aspx> |
| Neutral detergent fiber (NDF) | ANKOM Technology NDF Method 13 | A neutral detergent solution is used to dissolve the easily digested pectin and plant cell contents (proteins, sugars, and lipids), leaving a fibrous residue (NDF) that is primarily cell wall components of plants (cellulose, hemicellulose, and lignin). The percent NDF is determined from the weight lost following the extraction process relative to the dried sample weight.  <https://www.ankom.com/sites/default/files/document-files/Method_13_NDF_A2000.pdf> |

AOAC: Association of Official Analytical Chemists.
